# Use of a Caco-2 permeability assay to evaluate the effects of several Kampo medicines on the drug transporter P-glycoprotein

**DOI:** 10.1007/s11418-018-1222-x

**Published:** 2018-05-24

**Authors:** Takashi Matsumoto, Noriko Kaifuchi, Yasuharu Mizuhara, Eiji Warabi, Junko Watanabe

**Affiliations:** 1 Tsumura Kampo Research Laboratories, Kampo Research and Development Division, Tsumura & Co., 3586 Yoshiwara, Ami-machi, Inashiki-gun, Ibaraki 300-1192 Japan; 2 Division of Biomedical Science, Faculty of Medicine, University of Tsukuba, 1-1-1 Tennodai, Tsukuba-shi, 305-8575 Ibraki Japan

**Keywords:** Kampo medicine, Drug–drug interaction, Drug transporter, P-glycoprotein

## Abstract

In modern medical care in which Kampo and Western drugs are often combined, it is extremely important to clarify drug–drug interaction (DDI) to ensure safety and efficacy. However, there is little evidence of DDI in Kampo medicines. Therefore, as part of our studies to clarify the DDI risk for Kampo medicines, we evaluated the effects of five Kampo medicines [yokukansan (YKS), rikkunshito (RKT), shakuyakukanzoto (SKT), hangeshashinto (HST), and goshajinkigan (GJG)] that are widely used in Japan, on drug transporter P-glycoprotein (P-gp) using a Caco-2 permeability assay. These Kampo medicines inhibited the P-gp transport of digoxin through a Caco-2 cell monolayer. The IC_50_ values were 1.94–10.80 mg/ml. Of the five Kampo medicines, YKS showed the strongest inhibition (IC_50_ = 1.94 mg/ml), which was attributed to Uncariae Uncis Cum Ramulus. Unfortunately, we could not find the active ingredients responsible for its action. Finally, the *I*_gut_/IC_50_ values for the five Kampo medicines were calculated, and the DDI risk was objectively evaluated according to the criteria in the DDI guidance issued by the Japanese Ministry of Health, Labor, and Welfare and the US Food and Drug Administration. The *I*_gut_/IC_50_ values for the five Kampo medicines were ≤3.4. As these values were <10, they were evaluated as having a weak P-gp inhibitory effect that does not require further verification in humans, suggesting that the DDI risk due to P-gp inhibition for these Kampo medicines is low. The results should provide useful clinical information on the safety and efficacy of the combined use of Kampo and Western medicines.

**Electronic supplementary material:**

The online version of this article (10.1007/s11418-018-1222-x) contains supplementary material, which is available to authorized users.

## Introduction

Many reports have demonstrated that various metabolizing enzymes and transporters have important roles in the pharmacokinetics of xenobiotics [1–4]. P-glycoprotein (P-gp) is one of the major drug transporters expressed in different organs, including the small intestine, kidney, liver, lungs, colon, and blood–brain barrier [5, 6]. In the small intestine, P-gp is localized in the apical surface of intestinal epithelial mucosa and has the primary function of promoting removal of toxic compounds by active efflux back to the intestinal lumen [7]. Recently, venetoclax, which is indicated for the treatment of patients with chronic lymphocytic leukemia, enhanced the plasma concentration of co-administrated digoxin, a P-gp probe substrate, by inhibition of P-gp transporter [8]. Similarly, the levels of expression and functionality of P-gp can be modulated by inhibition or induction, which can affect the pharmacokinetics, efficacy, safety, or tissue levels of P-gp substrates, i.e., the safety and efficacy of drugs can be dramatically altered by drug–drug interactions (DDIs) which are influenced by the increase and decrease in P-gp activity.

Caco-2 cells derived from human colon carcinoma cells, which express P-gp and metabolizing enzymes such as peptidase, are widely used as an in vitro model of human small intestinal mucosa to predict the absorption of orally administered drugs [9] because of their morphological and functional similarity with human enterocytes [9, 10]. Digoxin is often used as a model P-gp substrate to assess transporter-mediated transport and inhibition in the Caco-2 permeability assay [11].

Many traditional Japanese Kampo medicines approved by the Japanese Ministry of Health, Labor, and Welfare (MHLW) have been used for the treatment of various diseases in Japan for many years. In modern medical care in which Kampo and Western drugs are often combined, it is important to clarify DDI to ensure the safety and efficacy of their combined use. However, there is little evidence of DDI in Kampo medicines. Yokukansan (YKS), rikkunshito (RKT), hangeshashinto (HST), goshajinkigan (GJG), and shakuyakukanzoto (SKT) are Kampo medicines which have been used for treating the behavioral and psychological symptoms of dementia, including aggression and insomnia [12], functional dyspepsia [13], stomatitis [14], and peripheral neuropathy [15], in patients with dementia and cancer or with muscle cramps [16]. Because the frequency of clinical use for these five prescriptions has recently increased rapidly, and opportunities to be used concomitantly with Western medicines have increased, safety information including drug interactions is required in medical practice. In order to promote the fostering and evolution of these Kampo medicines, we are actively accumulating scientific evidence to ensure their effectiveness and safety. Several studies on the DDIs or the side-effects of these five Kampo medicines have been reported previously, which include information on side-effects frequency surveys [17, 18] for YKS and SKT, cytochrome P-450 (CYP) [19] for YKS and RKT, and P-gp function using ATPase assay [20] or other drug transporter polypeptide 2B1 to organic anion-transporting polypeptide 2B1 [21] for various Kampo medicines. The results suggest that the possibility of causing severe pharmacokinetic DDIs through some types of drug-metabolizing enzyme and transporter or side-effects is low. However, to our knowledge, there is no evidence examining the effects of the five Kampo medicines on P-gp function using a Caco-2 permeability assay recommended by the Japanese Ministry of Health, Labor, and Welfare (MHLW) [22] and the US Food and Drug Administration (FDA) [23].

Therefore, in this study, the effects of five Kampo medicines (YKS, RKT, HST, GJG, and SKT) on P-gp drug transport were first examined. Then, the effects of the constituent crude drugs and some ingredients in YKS, which showed the strongest P-gp inhibitory effect, were further examined to clarify the contribution of YKS activity. Finally, the *I*_gut_/IC_50_ values for the five Kampo medicines were calculated, and the DDI risk was objectively evaluated according to the criteria in the DDI guidance of the MHLW [22] and FDA [23].

## Materials and methods

### Test substances and reagents

The biological activities of the five Kampo medicines and the composition of the constituent crude drugs are shown in Table 1. In the present study, the dry powdered extracts of YKS (Lot No. 321017700), RKT (Lot No. 332003900), HST (Lot No. 302149300), GJG (Lot No. 2120107020), and SKT (Lot No. 322098500), and seven crude drugs, constituting YKS {i.e., Uncariae Uncis Cum Ramulus (UUCR; Lot No. 2071089010), GR (Lot No. 281013010), Atractylodis Lanceae Rhizoma (ALR; Lot No. 2031005010), Poria (Lot No. 2031007010), Angelicae Acutilobae Radix (Lot No. 2031002010), Cnidii Rhizoma (Lot No. 2031004010), and Bupleuri Radix (Lot No. 2081020010)} were supplied by Tsumura & Co. (Tokyo, Japan). They were prepared by spray-drying a hot water extract from one crude drug or a mixture of several crude drugs. UUCR alkaloids geissoschizine methyl ether (GM) and rhynchophylline (RP) were obtained from AvaChem Scientific (San Antonio, TX, USA) and Tsumura & Co., respectively.

**Table 1:** Biological activity of the five Kampo medicines used in the present study and the composition of the constituent crude drugs

Kampo medicine	Known pharmacological activities	Constituent crude drugs (% composition)
Yokukansan	Amelioration of behavioral and psychological symptoms of dementia [12]	*Glycyrrhizae* radix (7.4), *Atractylodis lanceae* rhizoma (19.5), poria (19.5), cnidii rhizoma (14.6), *Uncariae* uncis cum ramulus (14.6), *Angelicae acutilobae* radix (14.6), bupleuri radix (9.8)
Rikkunshito	Improvement of upper gastrointestinal disorders [13]	*Atractylodis lanceae* rhizoma (18.6), *Ginseng* radix (18.6), *Pinelliae tuber* (18.6), poria (18.6), zizyphi fructus (9.3), citri unshiu pericarpium (9.3), *Glycyrrhizae* radix (4.7), *Zingiberis* rhizoma (2.3)
Shakuyakukanzoto	Amelioration of painful muscle cramps [16]	*Glycyrrhizae* radix (50), *Paeoniae* radix (50)
Hangeshashinto	Reducing chemotherapy-induced oral mucositis [14]	*Pinelliae tuber* (27.0), *Scutellariae* radix (13.5), *Zingiberis* rhizoma processum (13.5), *Glycyrrhizae* radix (13.5), zizyphi fructus (13.5), *Ginseng* radix (13.5), coptidis rhizoma (5.5)
Goshajinkigan	Reducing chemotherapy-induced peripheral neuropathy [15]	*Rehmanniae* radix (17.9), *Achyranthis* radix (10.7), corni fructus (10.7), *Dioscoreae* rhizoma (10.7), *plantaginis* semen (10.7), alismatis tuber (10.7), poria (10.7), moutan cortex (10.7), *Cinnamomi* cortex (3.6), *Aconiti* radix processa et pulverata (3.6)

[^3^H]-Digoxin (26.3–39.8 Ci/mmol), [^3^H]-mannitol (17.2 Ci/mmol), and ULTIMA Gold scintillation fluid were obtained from PerkinElmer Life and Analytical Sciences (Waltham, MA, USA). Digoxin was obtained from Alfa Aesar (Ward Hill, MA, USA). Mannitol was obtained from Wako Pure Chemical Industries (Osaka, Japan). Verapamil hydrochloride was obtained from Enzo Life Sciences (Farmingdale, NY, USA).

Fetal bovine serum, nonessential amino acids, penicillin, streptomycin, and l-glutamine used for Caco-2 cell culture were purchased from Thermo Fisher Scientific (Waltham, MA, USA).

Other chemicals were purchased from commercial sources.

### Test substance solutions

Various concentrations of the five Kampo medicines, seven crude drugs, and two ingredients (GM and RP) were prepared by dissolving in dimethyl sulfoxide (DMSO) or a mixture of DMSO and HEPES-buffered HBSS (HBSS-HEPES, pH 7.4) and then diluting with HBSS-HEPES buffer. The final concentration of DMSO was adjusted to ≤1% (v/v) in each study.

### Caco-2 cell culture

Caco-2 cells obtained from the American Type Culture Collection (Manassas, VA, USA) were routinely cultured in Dulbecco’s Modified Eagle Medium (DMEM; Thermo Fisher Scientific), including 10% fetal bovine serum, 1% nonessential amino acids, 100 U/ml penicillin, 100 µg/ml streptomycin, and 292 µg/ml l-glutamine, and incubated at 37 °C in an atmosphere with 5% CO_2_. They were split three times per week at a ratio of 1:4 upon reaching 80–90% confluence to use in the following cell viability and P-gp permeability assays. The cells used in this study were between 25 and 52 passages.

### Cell viability assay

The confluent Caco-2 cells (1.0 × 10^4^ cells/well) were seeded into 96-well plates and incubated for 2 days in the same constituent DMEM as described above. The media were then replaced with DMEM that included various concentrations of test substance or vehicle. Three or more wells were used for evaluation of each concentration. After the test substance-added wells were incubated for 2 h, the supernatant was removed, and 90 μl of HBSS-HEPES buffer and 10 μl of Cell Counting Kit-8 (Dojindo Laboratories, Kumamoto, Japan) were then added to the well. The cells were incubated for another 2–4 h at 37 °C in a CO_2_ incubator and measured at 450 nm using a Tecan Infinite M200 plate reader (Mannedorf, Switzerland). Cell viability was evaluated as a percentage of the absorbance of drug-treated cells relative to that of the vehicle-treated control (100% viability).

### Digoxin transport assay via P-gp

Caco-2 cells (2.7 × 10^4^ cells/insert) were seeded into a polyethylene terephthalate insert (0.4 mm pore size; Corning Life Sciences, Acton, MA, USA) in 24-well tissue culture plates. After culturing for approximately 21 days, the cell monolayer insert side was washed with warm phosphate-buffered saline (PBS), and the PBS in the apical chamber and the medium in the basolateral chamber were replaced with HBSS-HEPES buffer.

The transepithelial electrical resistance (TEER) to evaluate the integrity of the cell monolayer was measured using the Millicell ER-2 (Millipore, Billerica, MA, USA). For further verification of the tight junctions in the monolayer, warm HBSS-HEPES buffer containing mannitol (10 µmol/l) and [^3^H]-mannitol (0.058 µmol/l) was loaded into apical chambers, and the monolayer permeability of mannitol was measured in the same procedure as that of the following test substances. The integrity of a cell monolayer was confirmed by measuring the TEER, and mannitol permeability was used for evaluation of the test substance.

To evaluate the test substance effect on the permeability of digoxin via the cell monolayer, warm HBSS-HEPES buffer containing digoxin (10 µmol/l) and [^3^H]-digoxin (0.025–0.038 µmol/l) was added into either the apical or basolateral chamber in the absence or presence of various concentrations of test substances or verapamil (50 µmol/l). Aliquot solution in the apical or basolateral chamber was collected after incubation at 37 °C for 120 min with slow rotation in the horizontal direction and addition of an ULTIMA GOLD scintillation cocktail. The mixture was immediately injected into a liquid scintillation counter (AccuFLEX LSC-7200; Hitachi Aloka Medical, Tokyo, Japan) to measure the radioactivity.

The apparent permeability (*P*_app_) of digoxin was calculated as follows:

$$P_{\text{app}} = \frac{{\left( {\frac{{{\text{d}}Q}}{{{\text{d}}t}}} \right) \times V}}{{A \times C_{0} }}$$

where d*Q*/d*t* is the slope of the linear portion of the permeated amount versus time curve (µmol/l/s), *A* is the effective surface area of the Transwell insert (0.3 cm^2^), *C*_0_ is the initial concentration of the digoxin applied at *t* = 0 (µmol/l), and *V* is the volume of the receiver chamber (ml).

The efflux ratio to evaluate the P-gp inhibitory effect of the test substance was calculated as follows:

$${\text{Efflux ratio}} = \frac{{P_{{{\text{app}}, B \to A}} }}{{P_{{{\text{app,}} A \to B}} }}$$

where *P*_app, *A*→*B*_ is the apparent permeability of digoxin from the apical-to-basolateral direction and *P*_app, *B*→*A*_ is that from the basolateral-to-apical direction.

The inhibition ratios of P-gp by the test substances were determined as follows:

$${\text{Inhibition ratio }}(\% ) = ({\text{efflux ratio}}_{\text{test substance}} /{\text{ efflux ratio}}_{\text{control}} ) \times 100.$$

### Data analysis

The efflux ratios in each experiment were expressed as the mean ± standard deviation (SD). The statistical significance was evaluated by one-way analysis of variance (ANOVA) and Dunnett’s multiple comparisons test. A *P* value of < 0.05 indicated significance. IC_50_ values for P-gp inhibition of the five Kampo medicines were calculated by non-linear regression analysis using SAS 9.2 software (SAS Institute, Inc., Cary, NC, USA) or from the least square regression line. The concentration–response correlative formula and correlation coefficient (*R*^2^) for each crude drug constituting YKS were determined by regression analysis using Excel (Microsoft, Redmond, WA, USA).

## Results

### Cell viability

The viabilities of Caco-2 cells treated with various concentrations of test substances are summarized in Supplementary Table S1. From the results, the concentrations of test substances that showed little cell toxicity (i.e., cell viabilities >80%) were used in the digoxin transport assay using a Caco-2 cell monolayer.

### Effects of test substances on digoxin transport across a Caco-2 cell monolayer

The TEER value of the Caco-2 cell monolayer guarantees tight junction formation, which depends on the cell source, passage number, number of seeded cells, and culture conditions [24]. Based on this information, the TEER value corresponding to our experimental condition was judged to be ≥250 Ω cm^2^. Therefore, Caco-2 cell monolayers showing a TEER value of ≥250 Ω cm^2^ were first selected for this experiment. Next, the flux of mannitol across the cell membrane was examined to verify the integrity of the monolayer. The permeability of mannitol was < 1.5 × 10^−6^ cm/s, which indicated that the tight junctions of the cell monolayer were completed.

Furthermore, to confirm the expression and function of P-gp in the completed cell monolayer, transport of digoxin via the cell monolayer was investigated. The efflux ratio, an index for permeability of digoxin (6.98), was inhibited (0.832) by co-treating a P-gp inhibitor, verapamil (50 µmol/l). The inhibition rate was 88.1%, which shows that P-gp was expressed in the cell monolayer and functioned.

**Fig. 1:**
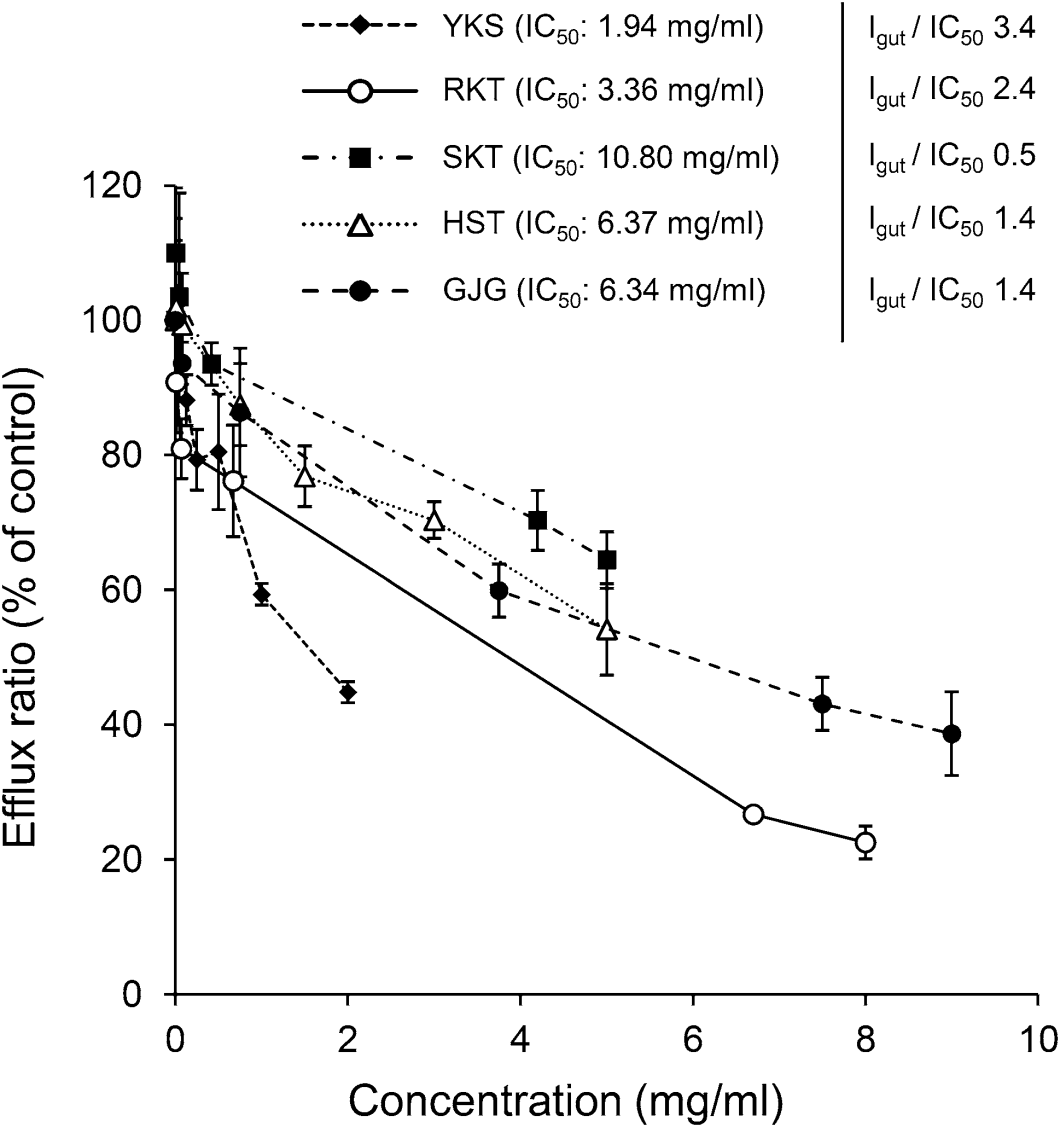
Inhibitory effects of five Kampo medicines on the efflux ratio across the Caco-2 cell monolayer of the P-gp substrate digoxin. Data represent the mean ± SD (*n* = 3–4). One-way ANOVA showed that each Kampo medicine significantly inhibited the efflux ratio in a concentration-dependent manner. The IC_50_ values for P-gp inhibition were calculated by non-linear regression analysis using SAS 9.2 software. The *I*_gut_/IC_50_ values were calculated as follows: *I*_gut_/IC_50_ = Inhibitor_dose in 250 ml_/IC_50_

Figure 1 shows the effects of the five Kampo medicines (YKS, RKT, SKT, HST, and GJG) on digoxin transport across the cell monolayer. All the Kampo medicines significantly inhibited the efflux ratio in a dose-dependent manner (YKS, *F*_5,18_ = 69.3, *P* < 0.001; RKT, *F*_5,12_ = 129.0, *P* < 0.001; SKT, *F*_5,12_ = 12.0, *P* < 0.001; HST, *F*_6,14_ = 35.2, *P* < 0.001; GJG, *F*_5,12_ = 32.0, *P *< 0.001). The IC_50_ values of YKS, RKT, SKT, HST, and GJG calculated by non-linear regression analysis were 1.94, 3.36, 10.80, 6.37, and 6.34 mg/ml, and the *I*_gut_/IC_50_ values as P-gp inhibition index were 3.4, 2.4, 0.5, 1.4, and 1.4, respectively. However, the IC_50_ values of SKT and HST were calculated as the estimated values, because both medicines did not show 50% inhibition in the examined concentration range.

**Fig. 2:**
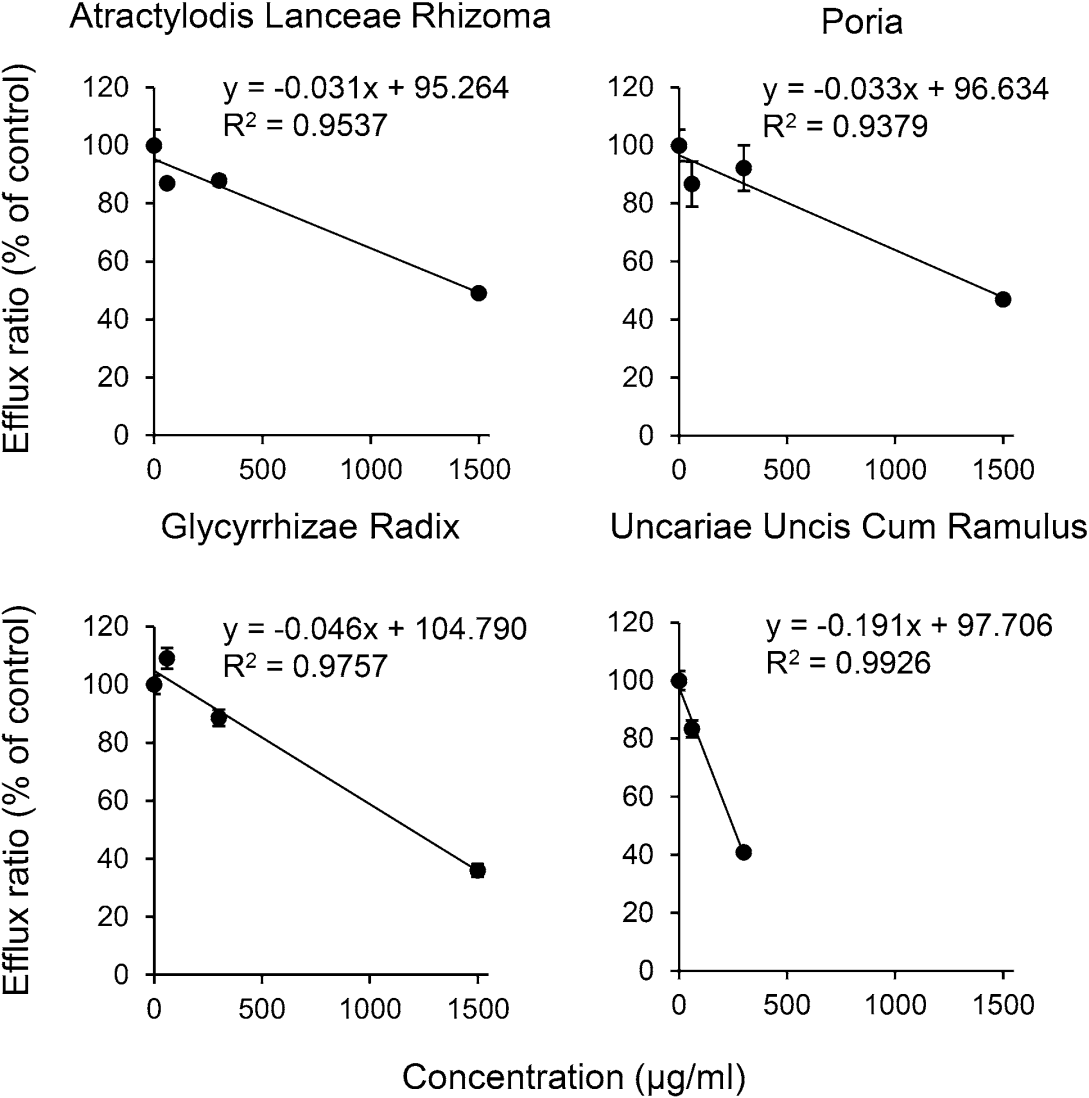
Inhibitory effect of four among seven crude drugs constituting YKS on the efflux ratio across a Caco-2 cell monolayer of the P-gp substrate digoxin. The dose–response correlative formula and correction coefficient (*R*^2^) of each crude drug were determined by regression analysis. Each data point represents the mean ± SD (*n* = 4). Concentration reactivity was statistically evaluated by one-way ANOVA

Next, the P-gp inhibition rates of seven crude drugs constituting YKS showing the strongest suppression of P-gp were examined. As shown in Fig. 2, four crude drugs (ALR, Poria, GR, and UUCR) among the seven constituents significantly inhibited the efflux ratio in a concentration-dependent manner (ALR, *F*_3,12_ = 229.0, *P* < 0.001; Poria, *F*_3,12_ = 58.4, *P* < 0.001; GR, *F*_3,12_ = 471.0, *P* < 0.001; UUCR, *F*_2,9_ = 577.0, *P* < 0.001). The correlativity was observed as *y* = − 0.031*x *+ 95.264 and the correlation coefficient as (*R*^2^) = 0.9537 for ALR, *y* = − 0.033*x* + 96.634 and *R*^2^ = 0.9379 for Poria, *y* = − 0.046*x* + 104.790 and *R*^2^ = 0.9757 for GR, and *y* = − 0.191*x* + 97.706 and *R*^2^ = 0.9926 for UUCR, where *y* is the efflux ratio and *x* is the drug concentration. From these formulas, the IC_50_ values were calculated to be 1,470 µg/ml for ALR, 1,430 µg/ml for Poria, 1,190 µg/ml for GR, and 249 µg/ml for UUCR. Other crude drugs (Angelicae Acutilobae Radix, Cnidii Rhizoma, and Bupleuri Radix) had low correlation, and the IC_50_ values could not be determined in the concentration range examined.

Table 2 shows the P-gp inhibition rate for each concentration of each constituent crude drug contained in the IC_50_ concentration (1.94 mg/ml, Fig. 1) of YKS to determine the contribution of each crude drug to the inhibitory effect of YKS. The concentration of each crude drug contained in the IC_50_ concentration of YKS was obtained from the composition ratio of the seven crude drugs constituting YKS. The inhibition rate at the concentration of each crude drug contained in the IC_50_ concentration of the YKS extract was calculated using the correlative formulas in Fig. 2. UUCR inhibited the majority (53.1%) of the total inhibitory activity of the seven crude drugs.

**Table 2:** The P-gp inhibition rate of seven crude drugs constituting YKS

Crude drug	Composition ratio of crude drug in YKS (%)	Concentration of crude drug in YKS (μg/ml)^a^	Contribution rate (%)^b^
*Uncariae* uncis cum ramulus	14.6	284	53.1
*Glycyrrhizae* radix	7.4	143	1.7
*Atractylodis lanceae* rhizoma	19.5	378	15.3
Poria	19.5	378	14.7
*Angelicae acutilobae* radix	14.6	284	3.0
Cnidii rhizoma	14.6	284	7.4
Bupleuri radix	9.8	189	4.8
Total	100.0	1,940	100.0

^a^Concentration of each crude drug in the IC_50_ concentration (1.94 mg/ml) of YKS extract was calculated from the composition ratio of seven crude drugs constituting YKS

^b^The contribution rate of each crude drug was calculated as a percentage of the total inhibition at each concentration included in the YKS (IC_50_ concentration) by using the correlative formulas described in result section and Fig. 2

**Fig. 3:**
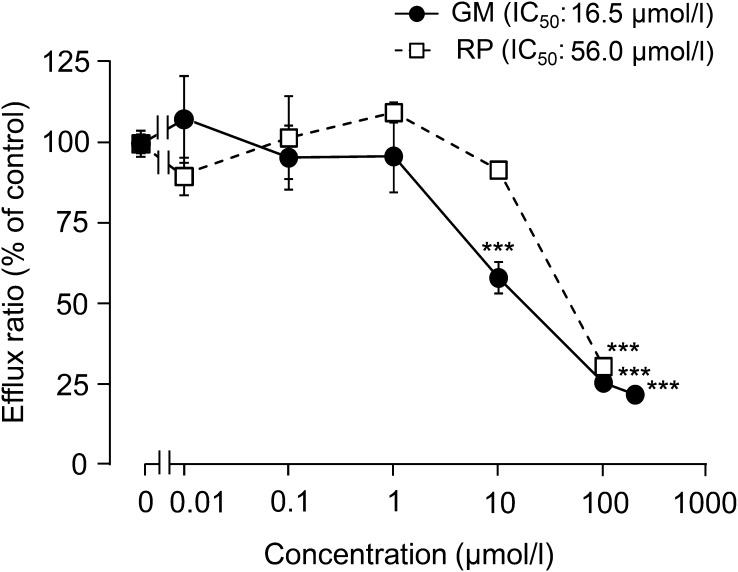
Inhibitory effects of GM and RP on the efflux ratio across the Caco-2 cell monolayer of the P-gp substrate digoxin. Each data point represents the mean ± SD (*n* = 3). ^***^P < 0.001 vs vehicle control: concentration reactivity was statistically evaluated by Dunnett’s test following one-way ANOVA

Next, the effects of GM and RP, which are UUCR alkaloids in YKS, were evaluated using a Caco-2 permeability assay (Fig. 3). GM (>10 μmol/l) and RP (100 μmol/l) significantly inhibited the efflux ratio (*P *< 0.001) compared to those in the control, and the IC_50_ values were 16.5 and 56.0 µmol/l, respectively.

The concentrations of GM and RP were 0.238 nmol/ml (0.238 µmol/l) and 0.179 nmol/ml (0.179 µmol/l) in the 1.94 mg/ml of YKS, respectively. To clarify the contributions of both ingredients to the inhibitory effect of YKS, although the inhibition rate at the concentration of each ingredient contained in the YKS extract were examined in Fig. 3, both ingredients were found to hardly inhibit P-gp at those concentrations (≤1.5% inhibitions).

## Discussion

We examined the inhibitory effect of the five Kampo medicines (YKS, RKT, SKT, HST, and GJG) widely used in clinical treatments for various diseases in Japan on P-gp transport of digoxin across a Caco-2 cell monolayer. Djuv and Nilsen [25] suggested the importance of measuring the cellular TEER, mannitol transport, and inhibition of verapamil on digoxin transport in a cultured cell monolayer to ensure the integrity of cell morphology and function in the Caco-2 permeability assay. In the present study, the monolayer, of which the integrity was confirmed by these indices, was used for evaluation of the test substance.

The P-gp transporter assay using a completed Caco-2 cell monolayer showed that all Kampo medicines examined inhibited the P-gp transport of digoxin, with YKS showing the strongest inhibitory action. Subsequent examination to clarify the contribution of the seven crude drugs constituting YKS showed that P-gp inhibitory action was observed in UUCR, GR, ALR, and Poria (Fig. 2). Although GR has been reported to suppress P-gp activity by ATPase assay [20], its contribution to the inhibitory activity of YKS was low as shown in Table 2. This result was also supported by other results, i.e., GR is included in four Kampo medicines (SKT, YKS, RKT, and HST) other than GJG. Of these GR-containing Kampo medicines, the GR content contained in SKT is 2–7 times greater than those of the other Kampo medicines. However, the P-gp inhibitory action of SKT was the weakest among the five Kampo medicines (IC_50_: 10.80 mg/ml). This result suggests that the GR level contained in each Kampo medicine may not contribute much to the P-gp inhibitory action of the five Kampo medicines, including YKS. On the other hand, UUCR is a crude drug that was contained only in YKS among the Kampo medicines examined in the present study, among which the inhibitory action of UUCR was stronger than the others (Fig. 2), and its contribution to the inhibitory activity of YKS was 53.1% (Table 2). Therefore, the potent P-gp inhibitory effect of YKS may be mainly due to the inhibitory action of UUCR, although the possibility that the effect may be due to additive or synergistic action with other crude drugs, such as GR, cannot be ruled out from this experiment.

We further investigated the P-gp inhibitory effect of the two major UUCR alkaloids (GM and RP) that were available for this study in order to to clarify the components contributing to the inhibitory activity of UCCR, and found that both ingredients had P-gp inhibitory activity. However, the P-gp inhibition rate at the GM and RP concentrations in the YKS extract powder was slight (≤1.5%), suggesting that other ingredients are involved in the inhibitory activity of UCCR. Because many other active alkaloids have been identified in YKS in addition to GM and RP [26], it is necessary to examine the P-gp inhibitory actions and additive/synergistic actions of other active alkaloids in the future. In addition, P-gp is also expressed in the luminal membrane of the blood–brain barrier, in the apical membranes of excretory cells, such as hepatocytes and kidney proximal tubule epithelia, and has an important role in the pharmacokinetics, efficacy, safety, or tissue levels of P-gp substrates [6]. We previously demonstrated that several UUCR alkaloids, including GM and PR, were detected in the plasma of rats treated with oral YKS [27]. It will be necessary to clarify the effect of these alkaloids on P-gp localized in other organs.

Both the Japanese MHLW [22] and US FDA [23] draft DDI guidance state that test drugs having an *I*_gut_/IC_50_ ≥10 are likely to be P-gp inhibitors. For test substances evaluated as P-gp inhibitors it is stated that more detailed DDI tests should be conducted in humans. Therefore, the *I*_gut_/IC_50_ values for the five Kampo medicines were calculated, and the DDI risk was objectively evaluated according to the criteria in the DDI guidance. The rates of YKS, RKT, SKT, HST, and GJG examined in the present study were 3.4, 2.4, 0.5, 1.4, and 1.4, respectively. These values were <10, which was evaluated as a weak P-gp inhibitory effect that does not require further verification in humans, suggesting that the DDI risk due to P-gp inhibition for these Kampo medicines may be low. Furthermore, it has been reported that the inhibitory potential of YKS and RKT against P-gp was low in an ATPase assay [28] and did not affect the plasma pharmacokinetics of digoxin in mice [19]. Ito et al. [19] and Soraoka et al. [29] also evaluated the effects of several Kampo medicines on CYP3A, which is known to have considerable overlap in substrate specificity and tissue localization of P-gp [30], and it has been reported that the inhibition rate of YKS on metabolic activity was low in mice and humans. Combining the above results, the P-gp inhibitory effect appears to be low in all five Kampo medicines examined in this study. In other words, in the present five Kampo medicines, it is thought that the possibility of drug interaction due to the inhibition of P-gp is extremely low in combination therapy with Western medicine.

## Conclusion

All Kampo medicines examined (YKS, RKT, SKT, HST, and GJG) inhibited the P-gp transport of digoxin in a Caco-2 permeability assay but to a low degree according to the criteria in the DDI draft guidance of the MHLW and FDA. These results suggest that the risk of drug interaction due to inhibition of P-gp in combination therapy with Western medicines is extremely low for these Kampo medicines. This finding should provide useful clinical information on the safety and efficacy of combinations of Kampo medicines and Western medicines.

## Electronic supplementary material

Below is the link to the electronic supplementary material.

Supplementary material 1 (DOCX 23 kb)
