# Supplementary material for: Use of a Caco-2 permeability assay to evaluate the effects of several Kampo medicines on the drug transporter P-glycoprotein
Source: J Nat Med. 2018 May 24;72(4):897–904. doi: 10.1007/s11418-018-1222-x (PMC6107777; doi:10.1007/s11418-018-1222-x)
Supplement: Supplementary file 1 — Supplementary material 1 (DOCX 23 kb) [file 11418_2018_1222_MOESM1_ESM.docx]

**Supplementary Table 1** Cell viability after test substances treatment.

| Experiment No. | Test sample | Concentration (µg/mL) | Cell viability (%) |
| --- | --- | --- | --- |
| Exp. 1 | Control | - | 100 ± 8.62 |
|  | Shakuyakukanzoto | 42 | 97.3 ± 7.23 |
|  |  | 420 | 97.7 ± 9.39 |
|  |  | 4200 | 95.6 ± 5.93 |
|  |  | 5000 | 81.5 ± 7.27 |
|  |  | 8000 | 60.9 ± 3.89 |
|  |  | 16000 | 46.4 ± 2.47 |
| Exp. 2 | Control | - | 100 ± 15.2 |
|  | Rikkunshito | 6.7 | 114 ± 3.82 |
|  |  | 67 | 118 ± 7.16 |
|  |  | 670 | 101 ± 4.55 |
|  |  | 6700 | 115 ± 5.91 |
|  |  | 8000 | 109 ± 6.6 |
| Exp. 3 | Control | - | 100 ± 9.69 |
|  | Hangeshashinto | 75 | 114 ± 3.63 |
|  |  | 750 | 105 ± 7.23 |
|  |  | 7500 | 76 ± 7.18 |
|  |  | 9000 | 60.5 ± 6.32 |
|  | Goshajinkigan | 75 | 94.9 ± 4.88 |
|  |  | 750 | 103 ± 6.37 |
|  |  | 7500 | 94.7 ± 9.02 |
|  |  | 9000 | 98.4 ± 5.45 |

continued

| Experiment No. | Test sample | Concentration (µg/mL) | Cell viability (%) |
| --- | --- | --- | --- |
| Exp. 4 | Control | - | 100 ± 19.3 |
|  | Yokukansan | 125 | 110 ± 4.13 |
|  |  | 250 | 106 ± 10.4 |
|  |  | 500 | 111 ± 9.28 |
|  |  | 1000 | 114 ± 5.12 |
|  |  | 2000 | 112 ± 2.58 |
|  | Angelicae Acutilobae Radix | 60 | 113 ± 3.95 |
|  |  | 300 | 118 ± 13.3 |
|  |  | 1500 | 129 ± 12.9 |
|  | Cnidii Rhizoma | 60 | 119 ± 11.4 |
|  |  | 300 | 113 ± 20.5 |
|  |  | 1500 | 115 ± 16.9 |
|  | Bupleuri Radix | 60 | 119 ± 5.58 |
|  |  | 300 | 124 ± 5.71 |
|  |  | 1500 | 145 ± 9.57 |
|  | Atractylodis Lanceae Rhizoma | 60 | 111 ± 14.5 |
|  |  | 300 | 101 ± 7.33 |
|  |  | 1500 | 104 ± 8.86 |
|  | Uncariae Uncis Cum Ramulus | 60 | 113 ± 8.55 |
|  |  | 300 | 99.5 ± 8.15 |
|  |  | 1500 | 36 ± 0.737 |
|  | Poria | 60 | 121 ± 2.62 |
|  |  | 300 | 120 ± 6.08 |
|  |  | 1500 | 106 ± 2.8 |
|  | Glycyrrhizae Radix | 60 | 104 ± 2.66 |
|  |  | 300 | 114 ± 9.07 |
|  |  | 1500 | 83.2 ± 3.48 |
| Exp. 5 | Control | - | 100 ± 4.16 |
|  | Geissoschizine methyl ether | 0.01 µmol/l | 92.8 ± 5.17 |
|  |  | 0.1 µmol/l | 104 ± 3.5 |
|  |  | 1 µmol/l | 106 ± 3.14 |
|  |  | 10 µmol/l | 101 ± 12.5 |
|  |  | 100 µmol/l | 92 ± 8.33 |
|  |  | 200 µmol/l | 104 ± 14.7 |
|  | Rhynchophylline | 0.01 µmol/l | 99.5 ± 4.86 |
|  |  | 0.1 µmol/l | 103 ± 6.13 |
|  |  | 1 µmol/l | 94.3 ± 3.63 |
|  |  | 10 µmol/l | 100 ± 3.39 |
|  |  | 100 µmol/l | 93.7 ± 5.69 |

Each data was represented the mean ± S.D. (n = 3–7).
